# Scaling Up Breastfeeding in Myanmar through the Becoming Breastfeeding Friendly Initiative

**DOI:** 10.1093/cdn/nzz078

**Published:** 2019-07-12

**Authors:** May Khin Than, Soe Nyi Nyi, Lwin Mar Hlaing, Swe Le Mar, Theingi Thwin, Jennifer Cashin, Rafael Pérez-Escamilla, Kassandra L Harding

**Affiliations:** 1 National Nutrition Centre, Department of Public Health, Ministry of Health and Sports, Nay Pyi Taw, Myanmar; 2 Save the Children, Bahan Township, Yangon, Myanmar; 3 Department of Medical Research, Ministry of Health and Sports, Nay Pyi Taw, Myanmar; 4 Alive & Thrive Southeast Asia, FHI 360, Yangon, Myanmar; 5 Yale School of Public Health, New Haven, CT, USA

**Keywords:** breastfeeding, breastfeeding gear model, policy, implementation, Myanmar

## Abstract

**Background:**

Optimal breastfeeding practices in Myanmar are above global averages, and the Ministry of Health and Sports (MoHS) has demonstrated its commitment to support nutrition and breastfeeding through continued policy and program actions. In 2017, the MoHS, in partnership with Save the Children, led the piloting of the Becoming Breastfeeding Friendly (BBF) Initiative. BBF provides a guide for countries to assess the enabling environment for breastfeeding and a country's readiness to scale up breastfeeding policies and programs.

**Objective:**

The aim of this study was to document the BBF process and outcomes in Myanmar.

**Methods:**

A Working Group (WG) of 14 members, led by a chair and 2 cochairs, conducted the BBF assessment using the BBF Index (BBFI), generated and prioritized recommendations, and disseminated the findings over the course of 5 meetings. Additional meetings were held to gain stakeholder endorsement and approval of the BBF process and WG before commencement and MoHS endorsement of the findings.

**Results:**

The BBFI score for Myanmar was 1.2 out of 3.0, which indicates a moderate environment for scaling up breastfeeding policies and programs. The Funding and Resources gear earned the lowest score (0.5), whereas Political Will earned the highest score (2.0). Overall, 4 gears were weak and 4 were moderate in strength. Nine recommendation themes were generated and prioritized. The top priority recommendation was to form a National Infant and Young Child Feeding Alliance. The MoHS endorsed the 9 recommendations in December 2018 and has provided leadership for the formation of the alliance.

**Conclusions:**

The BBF Initiative was successfully conducted in Myanmar, resulting in 9 prioritized recommendations for strengthening the breastfeeding enabling environment and substantial interagency collaborations. Adaptations to the BBF process were made for the context, and we note numerous lessons learned that should be considered by other countries that plan to commit to the BBF Initiative.

## Introduction

The benefits of breastfeeding for child survival, health, and development, as well as maternal health and well-being, have been well documented across low-, middle-, and high-income countries (1). By scaling up recommended breastfeeding practices to near-universal levels, an estimated 823,000 deaths among children <5 y of age in 75 low- and middle-income countries could be prevented each year, in addition to 20,000 maternal deaths due to breast cancer (1). Furthermore, breastfed children are at a reduced risk of becoming overweight or obese later in life, score better on intelligence tests, attain more years of schooling, and earn more income as adults (1). Breastfeeding also generates significant economic gains for households, communities, and countries. A study by Walters et al. (2) estimated US$1.6 billion in economic losses due to cognitive deficits associated with poor breastfeeding rates in 7 Southeast Asian countries, including Myanmar.

Despite the known benefits of breastfeeding, 3 out of 5 infants under 6 mo of age are not exclusively breastfed (3). Although nearly all women are biologically capable of breastfeeding, the decision to breastfeed is influenced by a variety of factors at the societal, community, household, and individual levels (4). National policies and programs have an impact on breastfeeding practices. Recent analysis of trend data from 38 low- and middle-income countries illustrates that increasing the duration of paid maternity leave is associated with a significantly higher prevalence of early initiation of breastfeeding, exclusive breastfeeding among infants under 6 mo, and longer duration of breastfeeding (5). Among countries with probreastfeeding social policies, breastfeeding rates have increased by 1% per year, or twice as fast as the global average (4).

In Myanmar, breastfeeding is near universal, with 98% of infants initiating breastfeeding (6). The exclusive breastfeeding rate has increased substantially in recent years from 23.6% among infants <6 mo of age in 2010 to 51.2% in 2016 (7). At 1 y of age, 87.9% of infants are still breastfeeding and at 2 y of age, 63.8% are still breastfeeding. The prevalence of early initiation of breastfeeding within 1 h of birth was estimated at 66.8% in 2016, representing a decline from 75.8% in 2010.

The Ministry of Health and Sports (MoHS), together with UNICEF and other partners, implemented a National Strategy for Infant and Young Child Feeding (IYCF) from 2011 to 2016 in order to improve, through recommended feeding, the nutritional status, growth and development, health, and survival of Myanmar children through a variety of interventions including behavior change communication, revitalization of the Baby Friendly Hospital Initiative (BFHI), and policy advocacy for breastfeeding (8). Major policy changes were also implemented during this period, with Myanmar signing up to the Scaling Up Nutrition (SUN) Movement in 2013, passing the Order of Marketing of Formulated Food for Infant and Young Child in 2014, and increasing the duration of paid maternity leave for the private sector (from 12 to 14 wk) and in the public sector (from 12 wk to 6 mo), also in 2014.

Since 2011, substantial political, economic, and administrative reforms have been achieved, with shifts to democratic governance and a market-based economy, which has resulted in increases in foreign investment and development assistance (9). Strong commitment to nutrition, as evidenced by the development of institutional governance mechanisms at the highest levels of government, has been an important enabling factor for improvements in nutrition behaviors and outcomes (10). Recognizing that breastfeeding is an essential component of the country's universal health care policy, Myanmar has included counseling on IYCF as a part of the Essential Health Package of Services under the National Health Plan (2018–2022) and explicitly included breastfeeding support in the new guidelines for antenatal care. Under the National Strategic Plan for Newborn and Child Health and Development (2015–2018), Myanmar has committed to increasing the coverage and quality of essential IYCF services and set ambitious targets for increasing the prevalence of exclusive breastfeeding among infants <6 mo to 60% and the early initiation of breastfeeding to 80% (11).

In order to identify concrete measures to scale up breastfeeding protection, promotion, and support programs and increase the country's breastfeeding rate, in March 2017, Myanmar committed to piloting the Becoming Breastfeeding Friendly (BBF) Initiative, and was the first country in Asia to do so (12). The first BBF assessment was conducted between January 2018 and August 2018. The objective of the current article is to document the BBF process and corresponding outcomes in Myanmar.

## Methods

BBF was developed by a research team at the Yale School of Public Health (12) based on the Breastfeeding Gear Model (BFGM) (13) and was pretested in Ghana and Mexico in 2016 (14–16). In brief, the BFGM posits that 8 “gears” must exist and work in harmony to generate an enabling environment for breastfeeding protection, promotion, and support. These gears include: *1*) Advocacy; *2*) Political Will; *3*) Legislation and Policies; *4*) Funding and Resources; *5*) Training and Program Delivery; *6*) Promotion; *7*) Research and Evaluation; and *8*) Coordination, Goals, and Monitoring. The BBF toolbox is designed to measure these gears and guide in the development and implementation of recommendations to address gaps within the gears. This occurs through a 5-meeting process carried out by a Working Group (WG) of breastfeeding stakeholders in the country, a BBF Index (BBFI) that is applied by the WG to score the breastfeeding environment, and case studies that support the scoring, recommendation development, and implementation processes. Myanmar joined a cohort of 8 countries to pilot BBF, and carried out the initial BBF 5-meeting process between January and August 2018.

### BBF WG formation and endorsement process

The MoHS and Save the Children developed a BBF WG that would be most effective for carrying out the BBF process in the Myanmar context. This included a chair (MKT), 2 cochairs (SNN and SLM), and 14 WG members (10 female) with expertise in breastfeeding and lactation, and represented the executive government branch (*n* = 12), parliament (legislative branch) (*n* = 1), nongovernment organizations (*n* = 2), and UN agencies (*n* = 2). This WG was presented to government stakeholders during a Stakeholder Endorsement Meeting for BBF, during which participants agreed upon the proposed WG composition and a work plan and provided approval for BBF to commence (**Supplemental Appendix 1**).

### The BBF 5-meeting process

**FIGURE 1:**
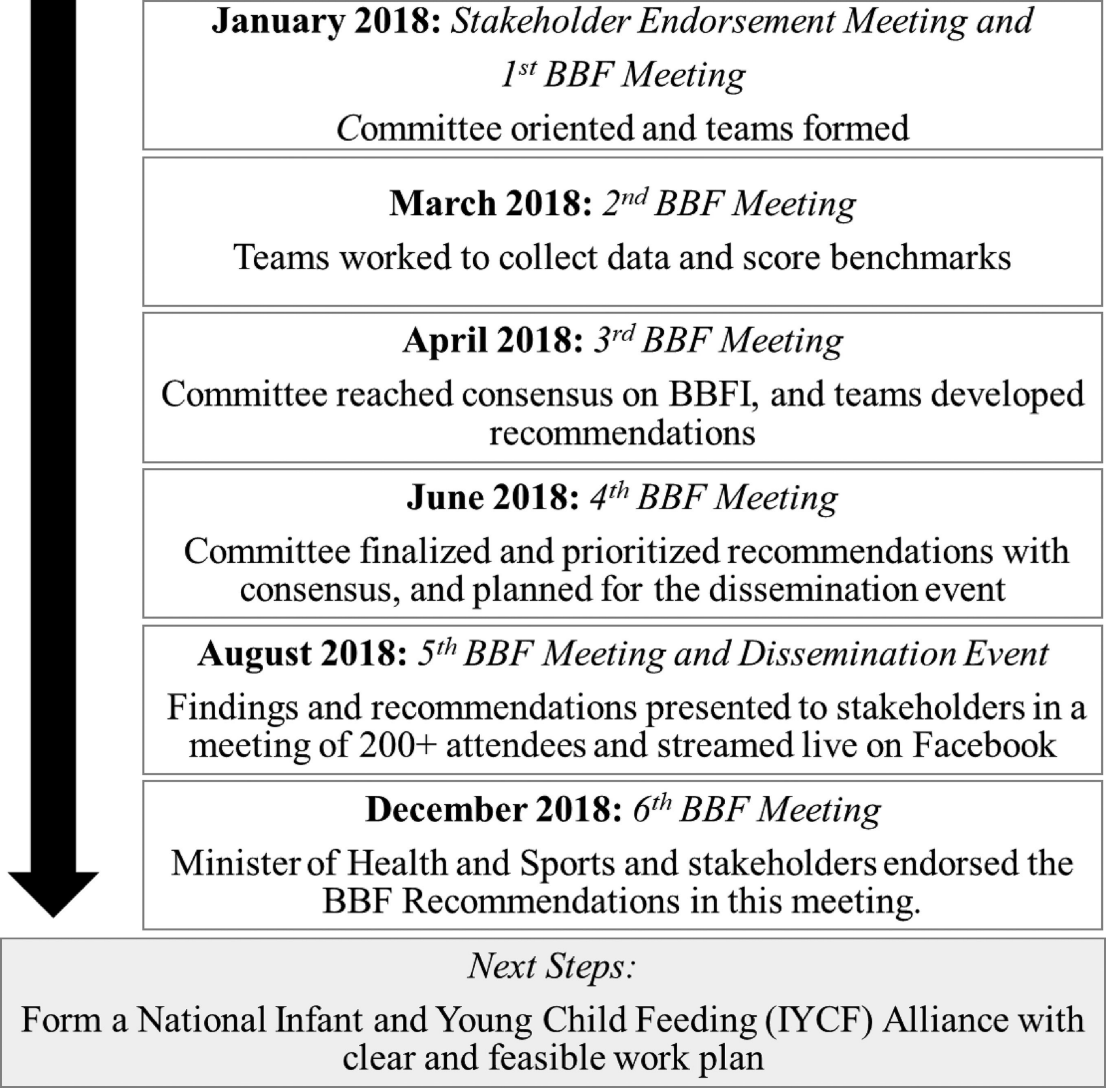
Timeline of BBF activities through the 6-meeting process. BBF, Becoming Breastfeeding Friendly; BBFI, Becoming Breastfeeding Friendly Index.

Immediately after the BBF Stakeholder Endorsement Meeting, the first BBF WG meeting was held (**Figure 1**). During this meeting, the chair and cochairs oriented the WG members on the BBF process and the BBFI. The BBFI consists of 54 benchmarks that measure the 8 gears of the BFGM. With the WG trained on the BBFI, they were assigned to teams that were each responsible for scoring benchmarks corresponding to 1–2 gears (**Table 1**). In the following 2 meetings, teams presented progress on the benchmark scores and gaps they had identified in the gears. The data collection involved reviewing policy documents, a media survey, interviews with key stakeholders, and reviewing reports from programs and surveys. By the third meeting, the WG used the Delphi Method to come to a consensus on the final scores.

**TABLE 1:** Characteristics of the 5 teams formed within the BBF WG^1^

Team	Number of WG members	Gear assignments	Number of benchmarks
1	3	Advocacy; Promotion	7
2	3	Legislation and Policies; Coordination, Goals, and Monitoring	13
3	3	Political Will; Funding and Resources	7
4	2	Research and Evaluation	10
5	3	Training and Program Delivery	17
Total	14	8 gears	54

1 BBF, Becoming Breastfeeding Friendly; WG, Working Group.

Benchmark scores were based on a 4-point scale: 0 = no progress, 1 = minimal progress, 2 = partial progress, and 3 = major progress. From the final benchmark scores, gear scores were calculated as the mean of the corresponding benchmark scores for that gear, as an indicator of gear strength based on the following cutoffs: 0 = gear not present, 0.1–1.0 = weak gear strength, 1.1–2.0 = moderate gear strength, and 2.1–3.0 = strong gear strength (**Table 2**). Gear scores were then weighted according to the BBF methodology (12). Each Advocacy gear score, Legislation and Policies gear score, Funding and Resources gear score, and Training and Program Delivery gear score was weighted 1.6; each Political Will gear score, Promotion gear score, and Research and Evaluation gear score was weighted 1.5; and the Coordination, Goals, and Monitoring gear score was weighted 1.4. To generate the total BBFI score, the weighted gear scores were totaled and divided by 12.3, the sum of all the gear weights. The BBFI score provides an indicator of the breastfeeding scale-up environment, with the following interpretations: 0–1.0 = weak scale-up environment, 1.1–2.0 = moderate scale-up environment, 2.1–2.9 = strong scale-up environment, and 3.0 = outstanding scale-up environment.

**TABLE 2:** Score interpretation^1^

Total gear score	Interpretation
0	Gear not present
0.1–1.0	Weak gear strength
1.1–2.0	Moderate gear strength
2.1–3.0	Strong gear strength

1 Gear scores were calculated as a mean of the corresponding benchmark scores presented in Supplemental Appendix 3 for a given gear, and categorized into 1 of 4 interpretations regarding the gear strength.

With the BBFI scoring finalized, the teams generated recommendations based on the scores and gaps for each of the gears they were responsible for. Recommendations across all gears were discussed by the full WG in the third meeting to consolidate the list. The chair and cochairs led a process of organizing the recommendations based on this WG discussion, and this list was presented at the fourth meeting for WG consensus. The WG then prioritized the recommendations through a 2-step process. In step 1, WG members completed a prioritization survey and graded each recommendation based on *1*) effectiveness, *2*) affordability, and *3*) feasibility using an adaptation of the Child Health and Nutrition Research Initiative research priority–setting methodology (17). In step 2, the WG discussed the survey results and reached a consensus on the final prioritization of recommendations.

After the recommendations were finalized and prioritized, the WG planned the dissemination policy event for the BBF findings and recommendations. The dissemination event was designed explicitly to generate attention around the BBF findings by both policy makers and the media, consistent with the BBF program impact pathways analysis (18). Therefore, local and national government representatives were invited, as well as major media outlets, and the event was streamed live on Facebook. A policy brief for the highest-priority recommendation and an infographic describing the BBF process and recommendations were generated by the WG and distributed at the meeting. The agenda consisted of presenting the BBF findings and recommendations, holding an engaging panel discussion to highlight key recommendations, and remarks by key officials to support the BBF recommendations (**Supplemental Appendix 2**).

This research was exempted from Yale University Institutional Review Board approval under the category of being a *public benefit or service program*, and was also exempted by the MoHS in Myanmar given the program implementation nature of the work.

## Results

The BBF chair, 2 cochairs, and 14 WG members were approved during the Stakeholder Endorsement Meeting in January 2018, and the BBF process commenced as planned and outlined in Figure 1 directly after this meeting. Of the 14 WG members, a mean ± SD of 89.5% ± 7.5% attended each of the initial 4 meetings during which the BBFI benchmarks were scored, and gaps and recommendations for the Myanmar breastfeeding environment were proposed, agreed upon, and prioritized (**Table 3**). Between the formal WG meetings, teams met a mean ± SD of 2 ± 2 additional times across the 9-mo period. At the fifth meeting, during which BBF findings and recommendations were disseminated to stakeholders, 6 of the 14 WG members attended, with absence due to the location and timing of the meeting. Based on guidance from the Union Minister for Health and Sports, the fifth meeting was held in Mandalay, which required at least a 4- or 8-h drive from Nay Pyi Taw or Yangon, respectively, where all WG members resided.

**TABLE 3:** Summary of the BBF meetings and WG member attendance^1^

Name of meeting	Meeting place	Date organized	WG member attendance (%)
Stakeholder Endorsement Meeting	Nay Pyi Taw	16 January, 2019	100
First BBF WG meeting	Nay Pyi Taw	16–17 January, 2019	100
Second BBF WG meeting	Nay Pyi Taw	21–22 March, 2018	88
Third BBF WG meeting	Nay Pyi Taw	27 April, 2018	76
Fourth BBF WG meeting	Nay Pyi Taw	21–22 June, 2018	94
Stakeholder endorsement on Scaling Up Breastfeeding in Myanmar (fifth BBF meeting)	Mandalay	27 August, 2018	41
Stakeholder endorsement on recommendations of BBF	Nay Pyi Taw	26 December, 2018	76

1 BBF, Becoming Breastfeeding Friendly; WG, Working Group.

**FIGURE 2:**
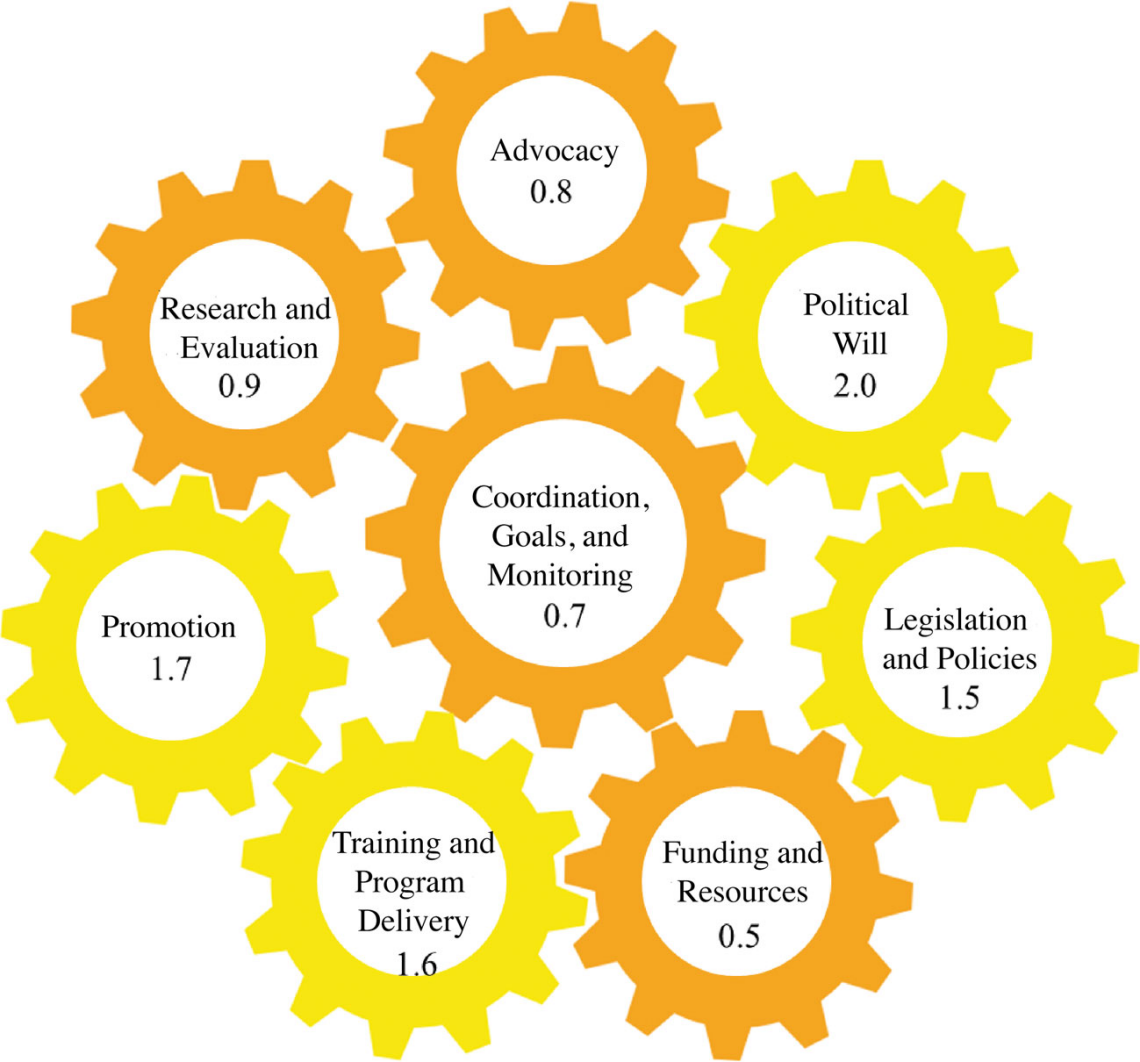
Final gear scores.

The overall BBFI score for Myanmar was 1.2, indicating a moderate environment for scaling up breastfeeding policies and programs. Gear scores ranged from 0.5 to 2.0, with 4 gears evaluated as weak and 4 gears as moderate in strength (**Figure 2**). In the following subsections, each gear is presented with the gear score and WG-proposed gaps (**Supplemental Appendix 3**).

### Advocacy gear

This gear was designed to measure the presence and activity of evidence-informed, community-driven advocacy for breastfeeding protection, promotion, and support, with an emphasis on 3 themes: *1*) public attention, *2*) individual champions, and *3*) social cohesion/mobilization. Four benchmarks were scored and averaged, yielding a gear score of 0.8.

A primary gap identified for this gear was the lack of a national advocacy strategy based on sound formative research. Only 1 breastfeeding advocate was identified during 2017 and WG members recognized minimal media coverage of IYCF and breastfeeding events. Most importantly, the WG found there was no formal, national cohesive network of advocates working to increase political and financial commitments to breastfeeding.

### Political Will gear

This gear measures policy makers’ expressed commitment to national breastfeeding scale-up efforts, with a single theme of expressed commitment measured by 3 benchmarks. The averaging of these benchmarks resulted in a gear score of 2.0, with the principal gap being a lack of public commitment to breastfeeding actions by political officials.

### Legislation and Policies gear

This gear measures the existence, coverage, and quality of policies and legislation that work to protect, promote, and support breastfeeding, with emphasis on 4 themes: *1*) national breastfeeding policy, *2*) the BFHI, *3*) the International Code of Marketing of Breast Milk Substitutes, and *4*) national maternity leave protection legislation. Ten benchmarks capture these 4 themes, and the gear score was 1.5.

National maternity leave protection and the Code were the themes with the greatest gaps. Whereas civil servants are entitled to 6 mo of maternity leave, the public sector is only entitled to 14 wk (6 wk before delivery and 8 wk postpartum). Furthermore, private sector employers are, in many cases, responsible for paying maternity leave benefits, and the existing maternity leave protection measures do not protect women from discrimination. Furthermore, the Factories Act (1951 rev. 2016) requires nursing spaces to be available for factory employees, yet this has not been enforced, and there is no existing legislation for nursing breaks or for requiring nursing spaces for nonfactory employees. With regards to the Code, Myanmar has implemented the Order of Marketing of Formulated Food for Infant and Young Child (the Order). However, not all provisions of the Code have been implemented. Breast-milk substitute companies can be granted exception on a case-by-case basis by the MoHS. Similarly to the Factories Act, the Order has not been enforced by the Technical WG cochaired by the National Nutrition Centre and the Food and Drug Administration. In recent years, Myanmar has revitalized the BFHI, with several of the country's leading maternity facilities being reassessed and designated as Baby Friendly since 2015, yet the standalone nature of the BFHI program remains a barrier to national scale-up and the reassessment of those certified hospitals for sustainable practice on the BFHI is yet to be established. In summary, maternity leave protection gaps include a lack of legislation for workplace lactation support (nursing breaks) as well as inadequate implementation coverage and enforcement of existing legislation on maternity protection (maternity leave and nursing spaces). Gaps related to the Code include enforcement of national legislation and gaps in the BFHI primarily relate to a lack of sustainability; reliance on external funding to support training, assessment, and monitoring; and therefore barriers to nationwide scale-up.

### Funding and Resources gear

This gear is designed to determine whether adequate funding and resources exist for the scaling up of breastfeeding programs, based on 4 benchmarks related to government budgetary commitment. This gear score was 0.8. Although there were some limitations to collecting the data required to score this gear, 2 primary gaps were identified: *1*) there is no national budget line for breastfeeding nor a government-funded position focused on breastfeeding, and *2*) there is no mechanism to monitor the provision of maternity entitlements.

### Training and Program Delivery gear

This is the most expansive gear, with the aim to assess whether adequate preservice and in-service training to support optimal breastfeeding exists for health workers, as well as the coverage and quality of facility- and community-based breastfeeding support programs. Seventeen benchmarks were scored to consider 9 themes: *1*) preservice training for health care providers, *2*) in-service training for facility-based providers, *3*) in-service training for community-based providers, *4*) in-service training for community health workers, *5*) train the trainers programs, *6*) coordination and integration of breastfeeding training programs, *7*) facility-based delivery of breastfeeding programs, *8*) community-based delivery of breastfeeding programs, and *9*) supervision of breastfeeding programs. Based on these themes and benchmarks, this gear score was 1.6.

With regards to the preservice training themes, the greatest gaps were in the consistent delivery of quality training and the inclusion of all essential breastfeeding topics and practical skills in training. There was an absence of breastfeeding master trainers at all levels and a recognized lack of consistent delivery of breastfeeding counseling, as well as a lack of coordination between training and program delivery. Dissemination of breastfeeding counseling guidelines across all facilities and to personnel providing maternity care was also limited.

### Promotion gear

This gear evaluates the presence and quality of promotional activities that support breastfeeding scale-up, emphasizing 2 themes: *1*) national breastfeeding promotion strategy, and *2*) government and civic breastfeeding promotion. Based on 3 benchmarks, we generated a gear score of 1.7 and identified 3 principal gaps. Although Myanmar did develop a National IYCF strategy, the strategy expired as of 2015 and therefore requires updating. There is also no framework in place to measure the effectiveness of the National IYCF promotion strategy, and finally, the WG recognized limited coverage of breastfeeding promotion on broadcast media.

### Research and Evaluation gear

This gear measures the presence of sound monitoring and evaluation systems designed to guide and assess national breastfeeding programs. Ten benchmarks are designed to consider 2 themes: *1*) breastfeeding outcomes and *2*) monitoring process indicators. This gear received a score of 0.9. Routine collection of data on key IYCF indicators, with the exception of early breastfeeding initiation, has not been incorporated into the Myanmar Health Management Information System and no breastfeeding data for vulnerable groups are available. Although the 2015–2016 Myanmar Demographic and Health Survey data and report are available on the MoHS website, many stakeholders are not aware of this data source.

### Coordination, Goals, and Monitoring gear

The Coordination, Goals, and Monitoring gear is considered the master gear of the BFGM and aims to assess the existence and operationalization of a government system responsible for coordinating national- and state-level breastfeeding programs. Three benchmarks are designed to evaluate the single theme of the existence and quality of such a system. Based on our findings, this gear received a score of 0.7 and 2 key gaps were identified. First, no breastfeeding committee exists in Myanmar to coordinate, guide, and support breastfeeding interventions. Second, nongovernment sources of data are rarely used for decision making and advocacy.

### Recommendations

Based on the data collection, benchmark scoring, and gap identification by the BBF WG, a total of 43 recommendations to fill the identified gaps were generated. These were narrowed down to 41 recommendations and grouped into 9 themes, with consensus from all WG members. Recommendations were prioritized according to the 2-step prioritization process described above (**Table 4**), and action plans and timelines were proposed for each recommendation (**Supplemental Appendix 4**). Of note, the top-priority recommendation was to form a National IYCF Alliance. Therefore, a 3-page policy brief specific to the formation of a National IYCF Alliance was developed by the WG for dissemination (**Supplemental Appendix 5**). Similarly, the overall findings and recommendations from the BBF process were distilled into a 2-page infographic, also for widespread dissemination (**Supplemental Appendix 6**).

**TABLE 4:** List of final recommendations in order of priority

Priority	Recommendation
1	Form a National Infant and Young Child Feeding Alliance with a clear and feasible work plan.
2	Mobilize a national cohesive network of advocates to develop and implement a national advocacy strategy.
3	Increase the availability and usage of breastfeeding data, including service delivery and prevalence of recommended practices, from the national to township level through the development of routine monitoring systems and through periodic household surveys.
4	Strengthen breastfeeding promotion through revising the communication strategy, developing standards for breastfeeding promotion and support, and increasing access and awareness through accessible media channels.
5	Update and strengthen preservice and in-service breastfeeding training for health providers and volunteers at community and health facility level, focusing on interpersonal counseling.
6	Increase the human resources allocated to supporting breastfeeding and providing certified lactation support.
7	Strengthen the implementation and coverage of the Baby Friendly Hospital Initiative through mandating the Ten Steps into hospital accreditation criteria.
8	Adopt full provisions of the International Breast Milk Substitutes Code and strengthen the monitoring and enforcement of The Order to more effectively regulate the marketing of breast-milk substitutes.
9	Revise paid maternity leave and protection legislation to include ≥6 mo for all sectors, clarify the terms of maternity leave, and protect pregnant and lactating women from workplace discrimination.

### Dissemination meeting and endorsement

The BBF process findings and recommendations were presented in a half-day dissemination event on 27 August, 2018 in Mandalay Region. A total of 142 people were in attendance, ranging from local health staff to the Prime Minister of Mandalay and Permanent Secretary of the MoHS. The Deputy Director of the National Nutrition Centre (LMH), and a BBF WG member, presented on the current breastfeeding situation in Myanmar, whereas the former Director of the National Nutrition Center, and chair of the BBF WG (MKT), presented the findings and recommendations from the BBFI. This was followed by a panel discussion entitled “Breastfeeding: Foundation of Life,” which included government officials, parliamentarians, medical doctors, and a famous novelist, who commented on BBF recommendations and findings. The event was also streamed live on Facebook via the Scaling Up Nutrition Civil Society in Myanmar page, with 1713 views across the 5 videos posted of the event.

After this event, the Deputy Director of the National Nutrition Center and WG members organized a sixth BBF meeting in Nay Pyi Taw entitled *Stakeholder Endorsement on Recommendations of Becoming Breastfeeding Friendly* on 26 December, 2018. The meeting was chaired by the Union Minister for Health and Sports. The meeting was attended by high-level officials from the MoHS such as the Union Minister, Permanent Secretary, and Director General as well as directors from relevant departments under the MoHS and WG members. Prioritized BBF recommendations were endorsed by the MoHS and stakeholders at this sixth meeting. As a priority action point, Terms of Reference for the National IYCF Alliance were requested to be submitted to the Union Minister for Health and Sports for approval. It is expected that the alliance will be formalized in the second half of 2019 or first half of 2020 as one of multiple technical working groups under the leadership of the National Nutrition Promotion Working Committee, a coordination mechanism agreed upon in the costed, Multi-Sectoral Plan of Action on Nutrition (July 2018), which is overseen by the Nutrition Promotion Steering Committee, chaired by the Union Minister for Health and Sports and including the Union Ministers for Social Welfare, Relief, and Resettlement, Education, and Agriculture, Livestock and Irrigation as members.

## Discussion

The overall BBF score for Myanmar was 1.2 out of 3.0, which represents a moderate environment for the scaling up of breastfeeding policies and programs. The strongest gear was Political Will with a score of 2.0 out of 3.0, and the weakest gear was Funding and Resources (0.5), followed by Coordination, Goals, and Monitoring (0.7). Although political commitment to improve breastfeeding and nutrition is strong, the lack of funding and institutions to effectively support the scale-up of breastfeeding programs is a weakness to address. Furthermore, Funding and Resources is consistently the weakest gear across countries who have scored the BBFI (14, 15), which may indicate a need for national and global revamping of budget priorities to support breastfeeding, and ultimately maternal and child health and nutrition.

According to the BFGM, the Coordination, Goals, and Monitoring gear is the “master gear” that allows a country's breastfeeding “engine” to work (13). As the WG synthesized the gaps across all gears, it was clear that a lack of coordination had weakened numerous gears, directly and indirectly. Thus, the top-priority recommendation was identified as “forming a National IYCF Alliance with a clear and feasible work plan.” The Terms of Reference for this alliance have been drafted and the alliance is expected to be formed under the leadership of the MoHS.

The Advocacy gear was also identified as weak in strength (score of 0.8), with the principal gap being the lack of a national advocacy strategy. This gear is needed to initiate a cascade that starts by activating political will, which in turn drives the stimulation of subsequent gears (13). Therefore, the second BBF priority recommendation was to mobilize a national cohesive network of advocates to develop and implement a national advocacy strategy. This should be incorporated in the work plan of the National IYCF Alliance once it is formalized.

In the BBF dissemination event (fifth meeting), the audience was receptive to the findings and recommendations. Since this event, a breastfeeding promotion campaign designed and implemented by Save the Children, Alive & Thrive, and UNICEF launched, the Myanmar BBF findings have been presented at the Breastfeeding: Advocacy and Practice Course organized by World Alliance for Breastfeeding Actions in Penang in September 2018 and at the General Assembly of SUN Civil Society Alliance Myanmar organized on 6 and 7 December, 2018, and the Minister of Health and Sports spoke of the importance of breastfeeding at the first meeting of the National Nutrition Steering Committee organized on 26 November, 2018 and attended by 3 other ministers from the Ministry of Agriculture, Livestock and Irrigation, the Ministry of Education, and the Ministry of Social Welfare, Relief and Resettlement, as well as social ministers from all states and regions.

The dramatic increase in the prevalence of exclusive breastfeeding in recent years has demonstrated that rapid change in behavior is possible in the Myanmar context. The BBF Initiative has provided concrete, evidence-based recommendations and action plans that can be referenced with confidence and acted upon by key players. This initiative has also resulted in unprecedented collaboration across government sectors and with nongovernmental organizations. As posited in the BBF impact pathways analysis, partnership building among WG members is a key process through which a country's WG can develop a collective agenda for advocacy, implement BBF recommendations, and build the country's capacity to strengthen the breastfeeding enabling environment (18).

Establishment of the National IYCF Alliance can be founded on the existing momentum built from BBF and the existence of WG members. Current WG members could be initial founding members of the alliance. Whenever possible, engaging with the Yale School of Public Health BBF Team for their inputs and support on development of Terms of Reference for the alliance, exchanging experiences from other countries, and documenting the learning process could be beneficial.

### Challenges in BBF implementation

Overall, BBF was feasible in Myanmar despite several challenges. The Government of the Union of Myanmar holds a strong commitment to improving nutrition and health across the country, which was an important facilitator to conducting BBF in Myanmar. There was a shortage in human resources for BBF both within the BBF leadership team and among WG members, who each had heavy workloads before the start of BBF. It proved challenging for WG members to commit to the 5 WG meetings as well as to interim gear team meetings. The WG leadership also experienced time pressures, as the chair and cochairs had full-time work and competing priorities apart from BBF. This was comparable with Ghana's experience, and in response to recommendations from Ghana's committee, the roles and responsibilities for WG members were explained in the WG member invitation and in the first meeting (14). Despite these explanations, it was still challenging for WG members to commit their time to BBF. It might be possible that the time challenges could be alleviated in part by *1*) identifying a full-time coordinator or cochair whose time is protected for managing and supporting BBF activities; and *2*) formalizing the commitment of WG members to BBF in a memorandum of understanding including expected time requirements. The specific working plan for the WG should be agreed upon a priori by, and clearly communicated to, the WG members, and gear chair and coordinators’ roles should be provided in writing.

Resource constraints, beyond human resources, were another challenge for Myanmar's BBF process. This work was primarily carried out with grant funding provided by the Yale School of Public Health. However, given the geographical dispersion of WG members, the need to provide honoraria to WG members, and changes in logistics, there were higher unforeseen expenses required to successfully carry out the BBF process. Cost sharing among partners had great advantages in addressing this resource constraint. Context-specific expenses must be considered by each country that plans to conduct BBF, and we recommend that countries considering BBF thoroughly consider from the beginning the resources required to complete BBF. Based on the aforementioned time demands of each person, we highly recommend that a BBF cochair or coordinator’s time be protected so that they may work full time managing and supporting the BBF activities.

An additional contextual consideration was the use of technology-based communication to organize and coordinate the BBF process. In-person communication was the most effective form of communication for the WG members given their busy schedules, as well as some variation in experience with different technology-based communication and file sharing platforms. Generally, BBF and interim meetings were conducted in person, and the cochairs and consultant developed deliverables and presentations to reduce the burden on WG members. However, this remained a limitation in instances when quick feedback by email was required. Other countries should develop a plan and expectations during the first BBF meeting regarding WG communications.

Finally, we faced some challenges with the outputs of the dissemination event. As described in the BBF impact pathways analysis, 2 key critical quality control points are *1*) the dissemination of recommendations and *2*) the policy makers’ reactions and media coverage of the recommendations (18). The dissemination event was organized in Mandalay, rather than Yangon or Nay Pyi Taw, in accordance with government guidance. Because of the location, there was a lower attendance among high-level officials, such as the Minister of Health and Sports, and the media engagement was not as strong as anticipated. As a result, additional opportunities were sought out for media promotion and coverage of the BBF recommendations, and an additional (sixth) meeting was held with the Minster of Health and Sports in order to gain endorsement for the findings and recommendations.

### Lessons learned

The BBF process presented a novel experience for those involved and generated significant learning. First, the leadership and collaboration were well-designed for Myanmar. The National Nutrition Center of the MoHS provided crucial leadership for BBF, which facilitated the engagement of other government departments and stakeholders. The National Nutrition Center leadership was supported by the Myanmar Scaling Up Nutrition Multi-Stakeholder Platform, which contributed to planning and logistics, liaising with partners, and supporting the implementation of BBF. Given that we were testing BBF in Myanmar and the methodology development was ongoing, guidance from the Yale School of Public Health was important for implementation of BBF. Second, the meeting locations were an important factor in Myanmar's context. All government WG members were based in Nay Pyi Taw, whereas most nongovernment WG members were based in Yangon. Holding meetings in Nay Pyi Taw ensured a greater level of attendance and participation from key government stakeholders and WG members. Third, we found it important to understand the methodology thoroughly to know where and how adaptations were appropriate. For instance, training videos that Yale shared for the WG members to watch were best viewed together during meeting time. Similarly, the recommendations’ prioritization was originally designed to be conducted through an online survey. However, for practical reasons we conducted it during a BBF WG meeting instead. Fourth, we designed a panel discussion for the dissemination event that included discussants from both the WG and outside the WG such as a famous writer, and a well-known OBGYN, a pediatrician, and female parliamentarians. This diverse panel was very well received, with positive feedback and engagement from the audience. Fifth, despite a media coverage plan and media organization attendance at the dissemination event, the media coverage was not as high as anticipated. In hindsight, we could have released a press release beforehand to support media dissemination of the event. Finally, it is crucial that the dissemination event is not viewed as the end of BBF. The BFF initiative is an ongoing commitment to strengthen the breastfeeding scale-up environment for programs and policies. Therefore, the dissemination event should be viewed as the starting point for implementing evidence-based recommendations and actions to improve national breastfeeding outcomes. The post–dissemination event momentum is a critical quality control point for the BBF initiative to consider moving forward.

### Conclusions

BBF was successfully completed in Myanmar with strong cooperation and coordination of partners, and yielded 9 evidence-based recommendations to strengthen the enabling environment for breastfeeding protection, promotion, and support. As a first step towards implementing these recommendations, we recommend the formation of an IYCF alliance and working plan which includes the development of policy briefs for all 9 recommendations and the translation of these into Burmese. Moving forward, we aim to support the implementation of these recommendations and monitor the country's progress towards the scale-up of breastfeeding programs and policies to ultimately improve maternal and child health and nutrition.

## Supplementary Material

nzz078_Supplement_Appendix

## Notes

Support for BBF comes from the Family Larsson-Rosenquist Foundation (FLRF) through an unrestricted grant to Yale University (principal investigator: RP-E). The contributions of Alive & Thrive were funded by Irish Aid.

Author disclosures: MKT, SNN, LMH, SLM, TT, JC, RP-E, and KLH, no conflicts of interest. We also disclose that the Family Larsson-Rosenquist owns the Medela breast pump company and that FLRF is run independently of the company.

Supplemental Appendices 1–6 are available from the “Supplementary data” link in the online posting of the article and from the same link in the online table of contents at https://academic.oup.com/cdn/.

Abbreviations used: BBF, Becoming Breastfeeding Friendly; BBFI, Becoming Breastfeeding Friendly Index; BFGM, Breastfeeding Gear Model; BFHI, Baby Friendly Hospital Initiative; IYCF, infant and young child feeding; MoHS, Ministry of Health and Sports; SUN, Scaling Up Nutrition; WG, Working Group.
