# Supplementary material for: Scaling Up Breastfeeding in Myanmar through the Becoming Breastfeeding Friendly Initiative
Source: Curr Dev Nutr. 2019 Jul 12;3(8):nzz078. doi: 10.1093/cdn/nzz078 (PMC6682606; doi:10.1093/cdn/nzz078)
Supplement: nzz078_Supplement_Appendix [file nzz078_supplement_appendix.zip › Appendix 1 - Stakeholder Meeting Agenda.pdf]

Becoming Breastfeeding Friendly (BBF)
Stakeholder Meeting

16 January 2018
Amara Hotel
Nay Pyi Taw, Myanmar

Final Agenda

**09:00 – 09:30**	**Welcome and Opening**	**Dr. Thaung Hlaing (DDG, PH)**
**09:30 – 09:45**	**Introduction**	**All Participants**
**09:45– 10:00**	**Tea Break**	
**10:00 – 10:30**	**Current Breastfeeding Situation and Background of BBF in Myanmar**	**Dr. May Khin Than (Director)**
**10:30 – 11:00**	**Introduction to BBF – Video Clip**	
**11:00 – 11:15**	**Stakeholder Engagement**	**Dr. Kassandra Harding, Yale University**
**11:15 – 11:30**	**Identification of Myanmar BBF Working Group Members**	**Dr. May Khin Than (Director)**
**11:30 – 11:45**	**Myanmar BBF Implementation Plan**	**Dr. May Khin Than (Director)**
**11:45 – 12:00**	**Discussion/Q and A**	
**12:00 – 12:15**	**Closing of the BBF Stakeholder Meeting**	
**12:15 – 13:00**	**Lunch Break**	

Note: BBF Committee Members' 1st Meeting will be started in the afternoon of the same day at 13:00 pm.
