# Supplementary material for: Scaling Up Breastfeeding in Myanmar through the Becoming Breastfeeding Friendly Initiative
Source: Curr Dev Nutr. 2019 Jul 12;3(8):nzz078. doi: 10.1093/cdn/nzz078 (PMC6682606; doi:10.1093/cdn/nzz078)
Supplement: nzz078_Supplement_Appendix [file nzz078_supplement_appendix.zip › Appendix 2 - Agenda for 5th meeting.pdf]

Stakeholder Endorsement on Scaling Up Breastfeeding in Myanmar

27 August 2018
Hotel Marvel, Mandalay

Proposed Agenda

08:30-09:00 Registration

09:00-10:00 Opening Speech
Dr. Zaw Myint Maung, Prime Minster, Mandalay Regional Government

09:30-10:00 Opening Speech
DyDG of MOHS on behalf of Minister of Ministry of Health and Sports

10:00-10:30 Tea Break

10:30-10:45 Current Situation on Breastfeeding in Myanmar
Dr. Lwin Mar Hlaing, Deputy Director, National Nutrition Center

10:45-11:00 Background of BBF
Dr. Kassandra Harding, Associate Research Scientist, Yale University

11:00-11:30 Findings and Recommendations of BBF in Myanmar
Dr. May Khin Than, Chair of the Country Working Group

11:30-12:30 Panel Discussion on "Breastfeeding: Foundation of Life"
Panelists:
- Dr. Kyi Kyi Nyunt, Prof. & Head (Retired), University of Medicine 2
- Dr. Yuzana Saw Myint, Associate Prof., University of Medicine 1
- Daw Khin Khin Htoo, Novelist
- Dr. Nwe Ni Ohn, Secretary, MMCWA
- Daw Aye Win, Director, Union Attorney General Office
- Dr. Tin Tin Win, Parliamentarian
- Dr. Khin Soe Soe Kyi, Parliamentarian
Moderator: Dr. Htin Lin, DyDG, FDA, MOHS

12:30-13:00 Discussion
13:00-13:05 Closing
13:05-14:00 Lunch
