# Supplementary material for: Scaling Up Breastfeeding in Myanmar through the Becoming Breastfeeding Friendly Initiative
Source: Curr Dev Nutr. 2019 Jul 12;3(8):nzz078. doi: 10.1093/cdn/nzz078 (PMC6682606; doi:10.1093/cdn/nzz078)
Supplement: nzz078_Supplement_Appendix [file nzz078_supplement_appendix.zip › Appendix 3 - Benchmark Scores.pdf]

Online Supporting Material 3. Scores for 54 Becoming Breastfeeding Friendly Index Benchmarks and 8 Gears

Gear	Benchmark Number	Benchmark	Score
Advocacy	AG1	There have been major events that have drawn media attention to breastfeeding issues.	1
	AG2	There are high-level advocates (i.e. 'champions') or influential individuals who have taken on breastfeeding as a cause that they are promoting.	1
	AG3	There is a national advocacy strategy based on sound formative research.	0
	AG4	A national cohesive network(s) of advocates exists to increase political and financial commitments to breastfeeding.	1
Advocacy Gear Score			*0.8*
Political Will	PWG1	High level political officials have publicly expressed their commitment to breastfeeding action.	1
	PWG2	Government initiatives have been implemented to create an enabling environment that promotes breastfeeding.	2
	PWG3	An individual within the government has been especially influential in promoting, developing, or designing breastfeeding policy.	3
Political Will Gear Score			*2*
Legislation and Policies	LPG1	A national policy on breastfeeding has been officially adopted/approved by the government.	3
	LPG2	There is a national breastfeeding plan of action.	2
	LPG3	The national BFHI/Ten Steps criteria has been adopted and incorporated within the healthcare system strategies/policy.	2
	LPG4	The International Code of Marketing of Breast Milk Substitutes has been adopted in legislation.	1
	LPG5	The National Code of Marketing of Breast Milk Substitutes has been enforced.	0
	LPG6	The International Labour Organization Maternity Protection Convention has been ratified.	2
	LPG7	There is paid maternity leave legislation for women.	3
	LPG8	There is legislation that protects and supports breastfeeding/expressing breaks for lactating women at work.	1
	LPG9	There is legislation for supporting worksite accommodations for breastfeeding women.	1
	LPG10	There is legislation providing employment protection and prohibiting employment discrimination against pregnant and breastfeeding women.	0
Legislation and Policies Gear Score			*1.5*
Funding and Resources	FRG1	There is a national budget line(s) for breastfeeding protection, promotion and support activities.	0
	FRG2	The budget is adequate for breastfeeding protection, promotion and support activities.	0

	FRG3	There is at least one fully funded government position to primarily work on breastfeeding protection, promotion and support at the national level.	0
	FRG4	There is a formal mechanism through which maternity entitlements are funded using public sector funds.	2
Funding and Resources Gear Score			*0.5*
Training and Program Delivery	TPDG1	A review of health provider schools and pre-service education programs for health care professionals that will care for mothers, infants and young children indicates that there are curricula that cover essential topics of breastfeeding.	1
	TPDG2	Facility-based health care professionals who care for mothers, infants and young children are trained on the essential breastfeeding topics as well as their responsibilities under the Code implementation.	3
	TPDG3	Facility-based health care professionals who care for mothers, infants and young children receive hands-on training in essential topics for counseling and support skills for breastfeeding.	2
	TPDG4	Community-based health care professionals who care for mothers, infants and young children are trained on the essential breastfeeding topics as well as their responsibilities under the Code implementation	2
	TPDG5	Community-based health care professionals who care for mothers, infants and young children receive hands-on training in essential topics for counseling and support skills for breastfeeding.	2
	TPDG6	Community health workers and volunteers that work with mothers, infants, and young children are trained on the essential breastfeeding topics as well as their responsibilities under the Code implementation.	2
	TPDG7	Community health workers and volunteers that work with mothers, infants, and young children receive hands-on training in essential topics for counseling and support skills for breastfeeding.	1
	TPDG8	There exist national/subnational master trainers in breastfeeding (i.e. breastfeeding specialists or lactation consultants) who give support and training to facility-based and community-based health care professionals as well as community health workers.	2
	TPDG9	Breastfeeding training programs that are delivered by different entities (e.g. face-to-face; on-line learning) through different modalities are coordinated.	1
	TPDG10	Breastfeeding information and skills are integrated into related training programs (e.g. maternal and child health, IMCI).	1
	TPDG11	National standards and guidelines for breastfeeding promotion and support have been developed and disseminated to all facilities and personnel providing maternity and newborn care.	2
	TPDG12	Assessment systems are in place for designating BFHI/Ten Steps facilities.	3
	TPDG13	Reassessment systems are in place to reevaluate designated Baby-Friendly/Ten Steps hospitals or maternity services to determine if they continue to adhere to the Baby Friendly/Ten Steps criteria.	1
	TPDG14	More than 66.6% of deliveries take place in hospitals and clinics designated or reassessed as “Baby- Friendly” in the last 5 years.	1
	TPDG15	Health facility-based community outreach and support activities related to breastfeeding are being implemented.	1

	TPDG16	Community-based breastfeeding outreach and support activities have national coverage.	1
	TPDG17	There are trained and certified lactation management specialists available to provide supportive supervision for breastfeeding program delivery.	1
Training and Program Delivery Gear Score			*1.6*
Promotion	PG1	There is a national breastfeeding promotion strategy that is grounded in the country's context.	1
	PG2	The national breastfeeding promotion strategy is implemented.	1
	PG3	Government or civic organizations have raised awareness about breastfeeding.	3
Promotion Gear Score			*1.7*
Research and Evaluation	REG1	Indicators of key breastfeeding practices are routinely included in periodic national surveys.	3
	REG2	Key breastfeeding practices are monitored in routine health information systems.	0
	REG3	Data on key breastfeeding practices are available at national and sub-national levels, including the local/municipal level.	2
	REG4	Data on key breastfeeding practices are representative of vulnerable groups.	0
	REG5	Indicators of key breastfeeding practices are placed in the public domain on a regular basis.	2
	REG6	A monitoring system is in place to track implementation of the Code.	1
	REG7	A monitoring system is in place to track enforcement of maternity protection legislation.	0
	REG8	A monitoring system is in place to track provision of lactation counseling/management and support.	0
	REG9	A monitoring system is in place to track implementation of BFHI/Ten Steps.	0
	REG10	A monitoring system is in place to track behavior change communication activities.	1
Research and Evaluation Gear Score			*0.9*
Coordination, Goals and Monitoring	CGMG1	There is a National Breastfeeding Committee/ IYCF Committee.	0
	CGMG2	National Breastfeeding Committee/IYCF committee work plan is reviewed and monitored regularly.	0
	CGMG3	Data/information related to breastfeeding program progress are used for decision-making and advocacy.	2
Coordination, Goals and Monitoring Gear Score			*0.7*

BFHI, Baby Friendly Hospital Initiative; IYCF, Infant and Young Child Feeding
Score interpretation: 0 = not done; 1 = minimal progress; 2 = partial progress; 3 = major progress
