# Supplementary material for: Scaling Up Breastfeeding in Myanmar through the Becoming Breastfeeding Friendly Initiative
Source: Curr Dev Nutr. 2019 Jul 12;3(8):nzz078. doi: 10.1093/cdn/nzz078 (PMC6682606; doi:10.1093/cdn/nzz078)
Supplement: nzz078_Supplement_Appendix [file nzz078_supplement_appendix.zip › Appendix 4 - Recommendations and Action Plan.pdf]

Prioritized Recommendations and Action Plan

Myanmar BBF

Contents

Prioritized Recommendation #1: Form a National IYCF Alliance with a clear and feasible work plan 2

Prioritized Recommendation #2: Mobilize a national cohesive network of advocates to develop and implement a national advocacy strategy 2

Prioritized Recommendation #3: Increase the availability and usage of breastfeeding data, including service delivery and prevalence of recommended practices, from the national to township level through the development of routine monitoring systems and through periodic household surveys 4

Prioritized Recommendation #4: Strengthen breastfeeding promotion through revising the communication strategy, developing standards for breastfeeding promotion and support, and increasing access and awareness through accessible media channels. 6

Prioritized Recommendation #5: Update and strengthen pre-service and in-service breastfeeding training for health providers and volunteers at community and health facility level, focusing on interpersonal counseling. 7

Prioritized Recommendation #6: Increase the human resources allocated to supporting breastfeeding and providing certified lactation support. 8

Prioritized Recommendation #7: Strengthen the implementation and coverage of BFHI through mandating the Ten Steps into hospital accreditation criteria 8

Prioritized Recommendation #8: Adopt full provisions of the International BMS Code and strengthen the monitoring and enforcement of The Order to more effectively regulate the marketing of breast milk substitutes 9

Prioritized Recommendation #9: Revise paid maternity leave and protection legislation to include at least 6 months for all sectors, clarify the terms of maternity leave, and protect pregnant and lactating women from workplace discrimination 9

Prioritized Recommendation #1: Form a National IYCF Alliance with a clear and feasible work plan

Sr.	Recommended Intervention	2018	2019	2020	2021	2022
1.1	National Alliance for Breastfeeding/IYCF needs to be formed under the Nutrition-specific TWG	x	x			
1.2	Work plan for implementation of BBF should be developed, reviewed and revised once the National Alliance for Breastfeeding/ IYCF is formed					
1.3	Strengthen multi-sectorial collaboration through Technical Working Group	x	x	x	x	
1.4	Strengthen the mechanism for sharing and accessing the data by the Technical Working Group (TWG)	x	x	x	x	

Prioritized Recommendation #2: Mobilize a national cohesive network of advocates to develop and implement a national advocacy strategy

Sr.	Recommended Intervention	2018	2019	2020	2021	2022
2.1	Engage a national cohesive network of advocates to increase political and financial commitments to breastfeeding at national, regional and local levels.					
2.1.1	Identify all potential networks (e.g., UN network, INGO network, Ministerial network, SUN CSA network, Local NGO network, Rice fortification network,..)	x				
2.1.2	Create the working groups representing the above networks at National, Regional and local levels and define roles and responsibilities	x				
2.1.3	Capacity building of National Advocacy Working Group members on Social Behavioral Change and Communication, advocacy, media coverage, campaign, and others	x	x	x	x	x
2.1.4	Conduct quarterly meetings of the working groups and produce detailed action plan		x	x	x	x

2.1.5	Implementation of the Detailed Action Plan		x			
2.1.6	Develop Monitoring and Evaluation plan		x	x	x	x
2.1.7	Continuous monitoring (Monthly)and evaluation (yearly)		x	x	x	x
2.2	Develop a national advocacy strategy, based on sound formative research, which targets high level policy makers at Ministerial level, parliamentarians and higher leaders. (Action: Media Coverage, Campaigns)					
2.2.1	Form national advocacy working group and work on strategy development	x				
2.2.2	Conduct quarterly meetings of the working groups and produce detailed action plan to create strategy		x	x	x	x
2.2.3	Hiring consultant for the desk review of the previous study and researches	x				
2.2.4	Form the formative research committee and accelerate the process of Ethical Review Committee clearance	x				
2.2.5	Conduct formative research nationally		x			
2.2.6	Develop national advocacy strategy targeting high level policy makers at Ministerial level, parliamentarians and higher leaders		x	x		
2.2.7	Champions lead in media coverage and campaign	x	x	x	x	x
2.3	Engage 3 or more champions who advocate for breastfeeding at least 3 times in a year.					
2.3.1	Find and choose 3 or more champions who advocate for breast feeding (e.g., Existing high level government staff can be a champion in advocating the minister or the State Counselor; Existing parliamentarian can be a champion in advocating the minister or the State Counselor)	x	x	x	x	x
2.3.2	Support the 3 or more breast feeding champions to be able to advocate for breastfeeding	x	x	x	x	x

Prioritized Recommendation #3: Increase the availability and usage of breastfeeding data, including service delivery and prevalence of recommended practices, from the national to township level through the development of routine monitoring systems and through periodic household surveys

Sr.	Recommended Intervention	2018	2019	2020	2021	2022
3.1	Develop an M & E system for the national Breastfeeding Program including internal monitoring system for BFHI and tracking BCC activities and BFHI/Ten steps in Government and Private Hospitals	x	x			
3.1.1	To Develop an M & E group for the national Breastfeeding Program	x	x			
3.1.2	To Develop an M & E framework for the national Breastfeeding Program	x	x			
3.1.3	Sustain implementation of national nutrition surveys which will include all key indicators for breast feeding and IYCF at least once in every five years and analyzing the causal factors for those KAPs.	x	x			
3.1.3.1	To conduct the national nutrition surveys assessing all key indicators of IYCF especially for Breastfeeding	x	x	x	x	x
3.1.4	Analysis for base-line information should be done for township level which could be available from DHS 2016.	x	x	x	x	x
3.1.4.1	DMR will analyze the existing breastfeeding MDHS 2016 data tentatively as ' The breastfeeding practices of mothers of under 2 year old children'	x	x			
3.1.5	Data from other sources, apart from Government sources, such as those from UN, NGOs and other researches should be used as appropriate for decision makings.	x	x			
3.1.5.1	To encourage and support the data dessimation of all avaiable suvery data	x	x			

Sr.	Recommended Intervention	2018	2019	2020	2021	2022
3.1.5.2	DMR will disseminate the survey data related to factors influencing initiation of breastfeeding among post- partum women from private clinics and hospitals in East district Yangon	x	x			
3.1.6	Supervision and monitoring on township level IYCF trainings by State/Region Nutrition Teams with special focus on those townships/districts which are resource limited.	x	x			
3.1.6.1	To implement supportive supervision program on township level IYCF trainings by State/Region Nutrition Teams with special focus on those townships/districts which are resource limited.	x	x			
3.1.6.2	To develop monitoring system IYCF trainings implementation	x	x			
3.1.7	Reporting system on in-service training to minimal information on existing capacity (e.g. no. of BHS trained) and gaps (SRNT to NNC).	x	x			
3.1.7.1	To develop reporting format/ record of in-service training to minimal information on existing capacity (e.g. no. of BHS trained) and gaps (SRNT to NNC).	x	x			
3.1.8	Developing internal monitoring group for BFHI activites in Government and Private Hospitals	x	x			
3.1.8.1	To develop internal monitoring group for BFHI activites in Government and Private Hospitals	x	x			
3.1.9	Developing and implementing the monitoring system for 3/5 yearly BFHI re-assessment	x	x			
3.1.9.1	To develop and implement the monitoring system for 3/5 yearly BFHI re-assessment	x	x			
3.1.1	To strengthen monitoring of breastfeeding support and maternal protection	x	x			

Prioritized Recommendation #4: Strengthen breastfeeding promotion through revising the communication strategy, developing standards for breastfeeding promotion and support, and increasing access and awareness through accessible media channels.

Sr.	Recommended Intervention	2018	2019	2020	2021	2022
4.1	Need to update the national breastfeeding promotion strategy grounded in country's context. The strategy has to be revitalized. (Breastfeeding specific communication strategy is needed)	x	x			
4.2	Standards & guidelines for breastfeeding promotion and support to be disseminated to all facilities & personnel providing maternity care.	x	x			
4.3	Increase access to breastfeeding promotion messages in rural areas with limited access to radio and TV by identifying and using accessible media channels (local media, social media, mobile platforms, etc.)	x	x			
4.3.1	Breastfeeding Song Competition through popular channels	x	x			
4.4	Need to raise awareness of the public by making IYCF information available on MOHS's website.	x	x			
4.5	Organize 3 or more major events that draw media attention to breast feeding issues at different times of the year like August NPM.	x	x			
4.6	Make sure campaigns and other advocacy activities have sound and impactful media coverage at the time of implementing the national advocacy strategy once it is developed.	x	x			

Prioritized Recommendation #5: Update and strengthen pre-service and in-service breastfeeding training for health providers and volunteers at community and health facility level, focusing on interpersonal counseling

Sr.	Recommended Intervention	2018	2019	2020	2021	2022
5.1	Update and strengthen pre service and in service IYCF trainings which include breastfeeding for health providers and volunteers at community and heath facility level, including focusing on interpersonal counselling	x	x	x		
5.1.1	Update IYCF training package to include health worker responsibilities under the Order of Marketing of Formulated Food for Infant and Young Child (FDA, NNC, ATGO)	x	x	x		
5.2	Form a National IYCF alliance with a clear and feasible work plan	x	x	x		
5.2.1	Supervision and monitoring on township level c-IYCF trainings by State/Region Nutrition Teams with special focus on those townships/districts which are resource limited	x	x	x		
5.2.2	Conduct C-IYCF trainings in all remaining State/Region levels (NNC)	x	x	x		
5.2.3	Regular Reporting system for number of IYCF counselling cases.	x	x	x		
5.3	Strengthen the implementation and coverage of BFHI through continuing advocacy of key steps of BFHI into Hospital accreditation criteria	x	x	x		
5.4	Strengthen monitoring system for BFHI re-assessment	x	x	x		
5.5	Strengthen coordination to hospital accreditation body	x	x	x		
5.6	Advocate standards & guidelines for breastfeeding promotion and support to be disseminated to all sectors, including public, private and military community, providing maternity care, etc.	x	x	x		

Prioritized Recommendation #6: Increase the human resources allocated to supporting breastfeeding and providing certified lactation support

Sr.	Recommended Intervention	2018	2019	2020	2021	2022
6.1	Human resources to support IYCF including breastfeeding to be rationally adequate at all levels.	x	x	x	x	x
6.1.1	Capacity need assessment?? To assign human resources for breastfeeding support at all levels	x	x	x	x	x
6.2	Establish the process to certify lactation management specialists/consultants??	x	x	x	x	x
6.2.1	Human resources; certified lactation consultants related to assess 24 hours breastfeeding counselling for special needs and specific breastfeeding problems and providing certified lactation support.	x	x	x	x	x

Prioritized Recommendation #7: Strengthen the implementation and coverage of BFHI through mandating the Ten Steps into hospital accreditation criteria

Sr.	Recommended Intervention	2018	2019	2020	2021	2022
7.1	Sustain and strengthen the implementation and coverage of BFHI.	x	x	x		
7.2	Strengthen monitoring system for BFHI re-assessment	x	x	x		
7.3	Strengthen coordination to hospital accreditation body	x	x	x		
8.4	Advocate standards & guidelines for breastfeeding promotion and support to be disseminated to all sectors, including public, private and military community, providing maternity care, etc.	x	x	x		

Prioritized Recommendation #8: Adopt full provisions of the International BMS Code and strengthen the monitoring and enforcement of The Order to more effectively regulate the marketing of breast milk substitutes

Sr.	Recommended Intervention	2018	2019	2020	2021	2022
8.1	Improve monitoring and enforcement of the Order of Marketing of Formulated Food for Infant and Young Child.	x				
8.2	Adopt full provisions of International BMS Code including new WHA resolutions in national legislation by removing loopholes and exceptional cases" from current national BMS order		x	x		
8.2.1	Paragraph 10 should be amended (the provision "with approval of the FDA or MOHS" should be omitted)		x	x		

Prioritized Recommendation #9: Revise paid maternity leave and protection legislation to include at least 6 months for all sectors, clarify the terms of maternity leave, and protect pregnant and lactating women from workplace discrimination

Sr.	Recommended Intervention	2018	2019	2020	2021	2022
9.1	Standardize maternity leave in all laws as at least six months for all sectors including public, and private including military community to align with WHO recommendations.	x	x			
9.1.1	Enforce the Social Security Law (Companies to register as required by the law)	x	x			
9.1.2	Update the Leave and Holidays Act in line with the ILO recommendations	x	x			

Sr.	Recommended Intervention	2018	2019	2020	2021	2022
9.1.3	Advocate the military community	x	x			
9.2	Clarify when maternity leave can be taken in order to ensure that mothers are not forced to stop working before delivery.	x	x			
9.2.1	Enforce the Social Security Law	x	x			
9.2.2	Advocate to revise the provision of section 11 of Social Security Law (especially in informal sector)	x	x			
9.2.2.1	For example - one year and above period for construction section should be amended. Shorten the duration of contract required.	x	x			
9.2.2.2	No provision for farming , livestock and planting sectors etc.	x	x			
9.3	Include protection from discrimination for pregnant and lactating women in maternity protection legislation.	x	x			
9.3.1	Need to enact a new law	x	x	x	x	x
9.4	Strengthen legislation for workplace breastfeeding policies by amending the Factory Act – Sub-section (1) of Section 50, Clause C of sub-section 2 of section 50 which demands employers for providing free formula milk or food or both.	x	x			
9.5	Provision of 'time, space, and support' for breastfeeding upon return to work, in line with ILO Recommendation 191.	x	x			
9.5.1	5.1. Issue the rules for nursing space and break in line with the Clause D of the sub-section 3 of the section 50 of the Factory Act	x	x			
9.6	Protection for a pregnant or nursing worker from engaging in work which could be detrimental to her health or that of her child, in line with ILO convention 183.	x	x			
9.6.1	Enforce the provision of the sub-section 3 and 4 of section 36 of the Factory Act	x	x			
