# Supplementary material for: Scaling Up Breastfeeding in Myanmar through the Becoming Breastfeeding Friendly Initiative
Source: Curr Dev Nutr. 2019 Jul 12;3(8):nzz078. doi: 10.1093/cdn/nzz078 (PMC6682606; doi:10.1093/cdn/nzz078)
Supplement: nzz078_Supplement_Appendix [file nzz078_supplement_appendix.zip › Appendix 5 - BBF Policy Brief.pdf]

Policy Brief: Myanmar

Becoming Breastfeeding Friendly

Policy Recommendation 1: Form a National Infant and Young Child Feeding Alliance

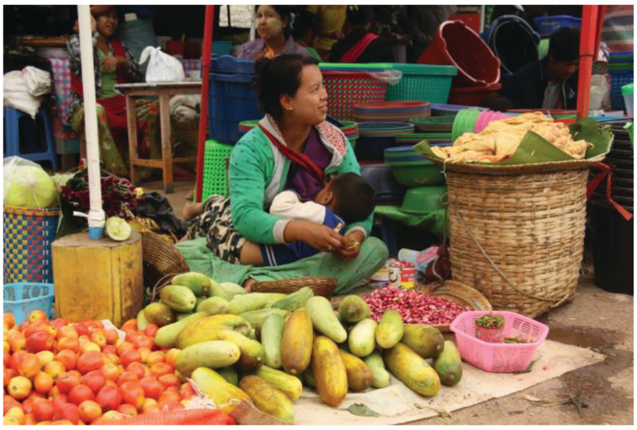

The scientific evidence is clear: breastfeeding gives children the best start in life. The World Health Organization (WHO) and the Myanmar Ministry of Health and Sports (MoHS) recommend exclusive breastfeeding for the first six months of a child's life, along with continued breastfeeding up to two years of age and beyond. Robust evidence shows that breastfeeding saves lives and directly supports health, social, and economic development – for individuals, communities and the nation (1, 2).

Who is this policy brief for?

Policy and decision makers, key stakeholders from government, civil society, UN, and donor networks

Why was it prepared?

'To address one of the key policy recommendations developed by the Becoming Breastfeeding Friendly (BBF) working group in Myanmar: **"Form a National Infant and Young Child Feeding Alliance"**

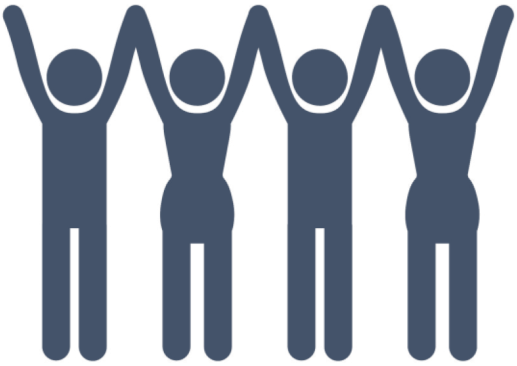

Supporting Breastfeeding Requires Leadership and Coordination from the Highest Levels of Government

Efforts to promote, protect, and support breastfeeding must involve and actively engage leaders within the health system, health workers, women's groups, medical associations, and policy makers to be successful. Efforts to scale up breastfeeding programs and initiatives cannot be effective without a strong, evidence-informed system for coordinating activities from the local to the national level. The 1990 Innocenti Declaration calls upon governments to appoint a national breastfeeding coordinator with authority to establish a multi-sectoral national breastfeeding committee comprised of representatives from relevant government departments and ministries, non-governmental organizations, and professional associations (3). At present, however, there is no breastfeeding coordinator in Myanmar and the national breastfeeding committee is not functional.

Policy Recommendation 1:
Form a National Infant and Young Child Feeding Alliance

Why does Myanmar need a National Infant and Young Child Feeding Alliance?

Global evidence shows that a functional National Breastfeeding Committee or Infant and Young Child Feeding Alliance is a critical step to scaling up breastfeeding programs. A well-functioning multi-sectoral committee or alliance can:

- Advocate for increased financial allocation and focus on breastfeeding as a development priority in Myanmar;
- Strengthen legislation to protect and support breastfeeding, such as the Order of Marketing of Formulated Food for Infant and Young Child and maternity protection benefits under the Social Security Law (4);
- Increase the coverage of effective interventions like the Baby Friendly Hospital Initiative (5);
- Increase the country's World Breastfeeding Trends Initiative (WBT*i*) score for national policy, programs, and coordination (5) and Global Breastfeeding Collective Country Scorecard (6);
- Improve infant and young child feeding practices (4).

Why is multi-sector representation important?

The National IYCF Alliance will aim to unite stakeholders, recognizing that interagency collaboration and coordination is critical to effectively promote, protect, and support breastfeeding (7). Representatives from relevant government ministries, UN agencies, civil society organizations, private companies, professional associations, and academic institutions should be involved in the National IYCF Alliance. Representation from non-health sectors such as education, labor, and social development should also be prioritized.

How much will it cost?

There are three cost components associated with the formation of a National IYCF Alliance.

Steps	Cost Components
**Step 1:**<br>**Recruitment and employment of a National Breastfeeding/ IYCF Coordinator**	• Annual salary;<br>• Benefit package; and<br>• Overhead costs such as office space, computer and accessories, telephone, internet, and email for communication along with travel-related costs.
**Step 2:**<br>**Mobilizing and formalizing the Alliance**	• Organizing meetings with potential members of the Alliance to agree on a Terms of Reference (TOR) including vision, mission, and objectives;<br>• Organizing quarterly meetings to develop and monitor the annual work plan.
**Step 3:**<br>**Sustaining the function and activities of the Alliance**	• Implementation of the annual work plan.*

* *The cost is dependent to the work plan of the Alliance. Government financing is preferred for sustainability, but funds from development partners engaged in the Scaling Up Nutrition Multi-Stakeholder Platform can also be mobilized.*

Action Steps: Forming a National IYCF Alliance to Improve Breastfeeding Rates, Save Lives, and Benefit the Economy

To scale up breastfeeding programs in Myanmar, the BBF Working Group calls on policy- and decision-makers from Parliament, Ministry of Finance and Planning, Ministry of Health and Sports, and Ministry of Labor, Immigration and Population, and the Attorney General's Office to:

- ❑ Nominate and appoint a National Breastfeeding Coordinator or a National Infant and Young Child Feeding Coordinator by the end of 2018, with a term of at least 2 years;
- ❑ Allow for and endorse the formation of a Multisectoral National Infant and Young Child Feeding Alliance including representatives from relevant government ministries, international and national civil society organizations and professional associations;
- ❑ Ensure that the Alliance develops and implements a work plan to coordinate and monitor the implementation of governmental and non-governmental programs and initiatives that protect, promote, and support breastfeeding;
- ❑ Provide financial support for the formation of the Alliance and sustaining its activities through human resources, including government staff participation.

References

Victora, C.G., Bahl, R., Barros, A.J.D., Franca, G.V.A., Krasevec, J., Murch, S., Sankar, M.J., Walker, N. & Rollines, N. C. for The Lancet Breastfeeding Series Group. (2016). Breastfeeding in the 21st century: epidemiology, mechanisms and lifelong effect. *Lancet 387*, 475 – 90.

Rollins, N.C., Bhandari, N., Hajeebhoy, N., Horton, S., Lutter, C.K., Martines, J.C., Piwoz, E.G., Richter, L.M. & Victora, C.G. on behalf of The Lancet Breastfeeding Series Group. (2016). Why invest, and what will it take to improve breastfeeding practices? *Lancet 387*, 491 – 504.

Innocenti Declaration on the Protection, Promotion and Support of Breastfeeding. Florence, Italy, WHO/UNICEF (Spedale degli Innocenti), 1990. Available at web site: http://www.unicef.org/programme/breastfeeding/innocenti/htm.

Yale School of Public Health. (2018). National Infant and Young Child Feeding (IYCF) Alliance of Bangladesh [Internet]. Available from: http://content.wkhealth.com/linkback/openurl?sid=WKPTLP:landingpage&an=00005650-200402001-00003

Yale School of Public Health. (2018). Brazil's Multi-Sectorial National Breastfeeding Committee [Internet]. Available from: http://content.wkhealth.com/linkback/openurl?sid=WKPTLP:landingpage&an=00005650-200402001-00003

UNICEF & WHO (2017). *Global Breastfeeding Scorecard 2017: Tracking Progress for Breastfeeding Policies and Programmes*. Myanmar country profile available from: https://www.unicef.org/nutrition/index_100585.html

US Department of Health and Human Services. (2011). *The Surgeon General's Call to Action to Support Breastfeeding.* Washington, DC: U.S. Department of Health and Human Services, Office of the Surgeon General.

Contact

Dr. May Khin Than, Chair
In-country BBF Working Group
maykt2011@gmail.com

Dr. Lwin Mar Hlaing, Acting Director
National Nutrition Center, DOPH, MOHS
lmhlaing78@gmail.com

Dr. Swe Le Mar, Co-chair/ Coordinator
In-country BBF Working Group
swelemar@gmail.com

Soe Nyi Nyi, Co-chair/ Coordinator
In-country BBF Working Group
soenyi.nyi@savethechildren.org

becoming breastfeeding friendly

A GUIDE TO GLOBAL SCALE-UP
