# Supplementary material for: Scaling Up Breastfeeding in Myanmar through the Becoming Breastfeeding Friendly Initiative
Source: Curr Dev Nutr. 2019 Jul 12;3(8):nzz078. doi: 10.1093/cdn/nzz078 (PMC6682606; doi:10.1093/cdn/nzz078)
Supplement: nzz078_Supplement_Appendix [file nzz078_supplement_appendix.zip › Appendix 6 - Infographic.pdf]

Becoming Breastfeeding Friendly Myanmar

The BBF Initiative guides countries in assessing current breastfeeding policies and practices and scaling up interventions to strengthen breastfeeding promotion, protection, and support.

*Key Recommendations From The 2018 Assessment

Form a National Infant and Young Child Feeding Alliance

Mobilize a cohesive national network of advocates to develop and implement a national advocacy strategy

Increase availability and utilization of breastfeeding data by developing routine monitoring systems and conducting periodic household surveys at sub-national and national level

Strengthen breastfeeding promotion by revising the communication strategy, developing breastfeeding promotion standards, and leveraging mass media

Update and strengthen lactation counseling during pre- and in-service training for health service providers and volunteers at facility and community levels, with a focus on interpersonal communication

Increase human resources allocated to support breastfeeding and provide certified lactation support

Strengthen implementation and coverage of the Baby Friendly Hospital Initiative by mandating the inclusion of key elements of the Ten Steps into hospital accreditation

Adopt full provisions of the International Code of Marketing of Breastmilk Substitutes and strengthen monitoring and enforcement of the Order of Marketing of Formulated Food for Infant and Young Child

Revise legislation for maternity leave and protection to include at least six months of paid leave in all sectors, clarify the terms of maternity leave, and protect pregnant and lactating women from workplace discrimination

'Breastmilk is the first food for babies. Breastfeeding can reduce family expenditures on health care as babies are healthier. At the policy level, breastfeeding contributes significantly to national development. Thus, we need to encourage breastfeeding. ILO, according to Maternity Protection Convention 183, recommends longer duration of maternity leave.' - Dr. Myint Htwe, Union Minister, Ministry of Health and Sports (MoHS)

bbf becoming breastfeeding friendly A GUIDE TO GLOBAL SCALE-UP | Yale SCHOOL OF PUBLIC HEALTH | Ministry of Health and Sports | Save the Children | Scaling Up Nutrition | Civil Society Alliance Myanmar | alive&thrive nourish.nurture.grow

Becoming Breastfeeding Friendly

Breastfeeding in Myanmar

In Myanmar, exclusive breastfeeding among infants under six months of age increased from 23.6% in 2010 to 51.2% in 2016, demonstrating the country's potential to rapidly improve life-saving nutrition behaviors. Despite progress, however, there is still work to be done: early initiation of breastfeeding within one hour of birth decreased from 75.8% to 66.8% during the same period and continued breastfeeding and one and two years of age also decreased slightly. [1, 2]

The Ministry of Health and Sports (MoHS) is committed to facilitating an enabling environment for breastfeeding. MoHS has promoted WHO recommended breastfeeding practices by adopting the International Code of Breastmilk Substitutes, implementing the Community Infant and Young Child Feeding counseling package, expanding the Baby Friendly Hospital Initiative, and providing longer maternity leave for civil servants in collaboration with other stakeholders. Most recently, MoHS led the Becoming Breastfeeding Friendly (BBF) Initiative, launched in Myanmar in January 2018.

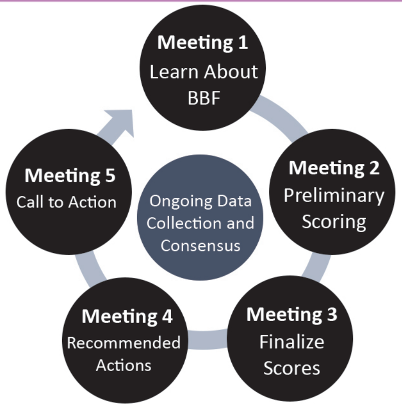

Figure 1. BBF Five-Meeting Process

BBF in Myanmar

A working group of 17 health and nutrition experts convened to implement and support the BBF initiative. Becoming Breastfeeding Friendly (BBF) assists countries to:

- measure the current breastfeeding-friendly environment; and
- develop a plan and recommendations to guide the scale up of national breastfeeding protection, promotion, and support efforts.

BBF provides countries with a toolbox that includes a 54-benchmark indices to assess the 8 gears in the Breastfeeding Gear Model, as well as case studies and a five-meeting process that guides countries through the process of scaling up breastfeeding (Figure 1).[3]

BBF Findings in Myanmar

- The overall breastfeeding scale-up environment was identified as moderate, with a score of 1.2 (see Table 1 for interpretation).
- Funding & Resources, Coordination, Goals & Monitoring, Advocacy, and Research & Evaluation were identified as the weakest processes with the lowest scores.
- Political will is the best performing gear with the highest score of 2.0, ranking as moderate strength (Figure 2).
- Nine overarching recommendations were developed and prioritized by the country working group based on the Myanmar context and BBF findings (see reverse side).

Table 1. Score interpretations

Gear Total Score	Interpretation
0	Gear not present
0.1 to 1.0	Weak Gear Strength
1.1 to 2.0	Moderate Gear Strength
2.1 to 3.0	Strong Gear Strength

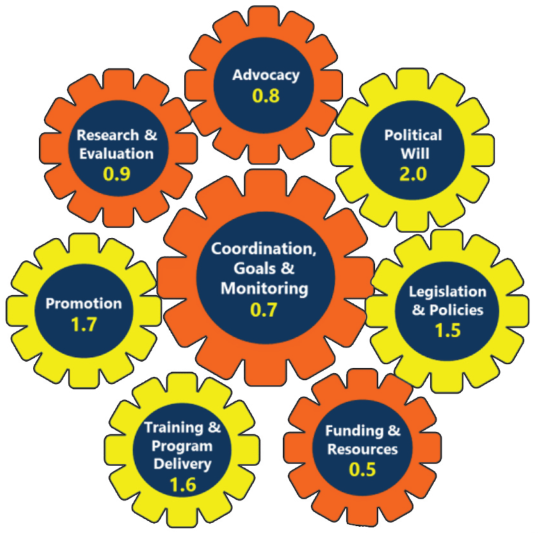

Figure 2. Myanmar 2018 BBF Index Gear Scores*

Contact to Learn More:

Dr. May Khin Than, Chair
In-Country BBF Working Group
maykt2011@gmail.com

Dr. Lwin Mar Hlaing , Acting Director
National Nutrition Center,
Ministry of Health and Sports
lwinmarhlaing@mohs.gov.mm

References

[1] Ministry of National Planning and Economic Development & Ministry of Health. (2011). Myanmar *Multiple Indicator Cluster Survey 2009 – 2010 Final Report*. Nay Pyi Taw, Myanmar: Ministry of National Planning and Economic Development & Ministry of Health.

[2] Ministry of Health and Sports (MoHS) and ICF. (2017). *Myanmar Demographic and Health Survey 2015-16*. Nay Pyi Taw, Myanmar, and Rockville, Maryland USA: Ministry of Health and Sports and ICF.

[3] Pérez-Escamilla, R., Hromi-Fiedler, A.J., Gubert, M.B., Doucet, K., Meyers, S. & dos Santos Buccini, G. (2018).Becoming Breastfeeding Friendly Index: Development and application for scaling-up breastfeeding programmes globally. *Matern Child Nutr. 2018;14:e12596.*
